# Rare variant phasing using paired tumor:normal sequence data

**DOI:** 10.1186/s12859-019-2753-1

**Published:** 2019-05-27

**Authors:** Alexandra R. Buckley, Trey Ideker, Hannah Carter, Nicholas J. Schork

**Affiliations:** 1 Biomedical Sciences Graduate Program, University of California, San Diego, La Jolla, CA USA; 2 Human Biology Program, J. Craig Venter Institute, La Jolla, CA USA; 3 Department of Quantitative Medicine and Systems Biology, The Translational Genomics Research Institute, Phoenix, AZ USA; 4 Departments of Family Medicine and Public Health and Psychiatry, University of California San Diego, La Jolla, CA USA; 5 Division of Medical Genetics, Department of Medicine, University of California San Diego, La Jolla, CA USA; 6 Moores Cancer Center, University of California San Diego, La Jolla, CA USA; 7 Cancer Cell Map Initiative (CCMI), University of California San Diego, La Jolla, CA USA

**Keywords:** Variant phasing, Cancer germline, Cancer genomics

## Abstract

**Background:**

In standard high throughput sequencing analysis, genetic variants are not assigned to a homologous chromosome of origin. This process, called haplotype phasing, can reveal information important for understanding the relationship between genetic variants and biological phenotypes. For example, in genes that carry multiple heterozygous missense variants, phasing resolves whether one or both gene copies are altered. Here, we present a novel approach to phasing variants that takes advantage of unique properties of paired tumor:normal sequencing data from cancer studies.

**Results:**

VAF phasing uses changes in variant allele frequency (VAF) between tumor and normal samples in regions of somatic chromosomal gain or loss to phase germline variants. We apply VAF phasing to 6180 samples from the Cancer Genome Atlas (TCGA) and demonstrate that our method is highly concordant with other standard phasing methods, and can phase an average of 33% more variants than other read-backed phasing methods. Using variant annotation tools designed to score gene haplotypes, we find a suggestive association between carrying multiple missense variants in a single copy of a cancer predisposition gene and earlier age of cancer diagnosis.

**Conclusions:**

VAF phasing exploits unique properties of tumor genomes to increase the number of germline variants that can be phased over standard read-backed methods in paired tumor:normal samples. Our phase-informed association testing results call attention to the need to develop more tools for assessing the joint effect of multiple genetic variants.

**Electronic supplementary material:**

The online version of this article (10.1186/s12859-019-2753-1) contains supplementary material, which is available to authorized users.

## Background

Humans have two copies of every chromosome, one inherited maternally and the other paternally. Assigning genetic variants to their homologous chromosome of origin is called phasing. Studying genetic variants in the context of their phased haplotype, termed diplomics, can yield important biological insights [1]. There are three main strategies for phasing variants in unrelated individuals using next generation sequencing (NGS) data: population-based, which relies on population linkage disequilibrium structure, laboratory-based, which relies on physical isolation of homologous chromosome segments, and read-backed, which relies on paired-end sequencing reads that span multiple heterozygous loci [1, 2]. Each method comes with a cost: population-based methods perform poorly on rare and de novo variants and at phasing distances greater than a haplotype block, laboratory-based methods require sample preparation which can be costly or impractical depending on the source of input DNA, and read-backed methods generally can only phase a fraction of possible variants at distances limited by read and insert size.

Here we present VAF phasing, a method that uses changes in variant allele frequency (VAF) between paired tumor and normal samples in regions of somatic chromosomal copy loss or gain to phase germline variants. Similar to read-backed approaches, VAF phasing requires only NGS data, and can phase both common and rare variants. Unlike read-backed approaches, VAF phasing is not limited by read and insert size, and can phase over long distances including whole chromosomes. VAF phasing is limited to regions of somatic copy number alteration (SCNA); however, SCNAs are widespread in cancer and approximately 90% of solid tumors exhibit some degree of aneuploidy [3]. The concept of using allelic ratios of heterozygous germline variants to infer somatic copy number changes is the basis for many SCNA detection algorithms [4, 5]. However, using this data to infer the phase of germline variants has not been widely implemented. While a similar method of using SCNAs to phase germline variants exists, VAF phasing is a more simple approach that can be run without training data [6].

There is growing interest in the role of germline variation in increasing cancer risk and influencing molecular tumor phenotypes [7–10]. Many cancer-relevant genes, such as DNA damage repair genes, are large and often contain multiple missense variants [11, 12]. Phasing damaging heterozygous variants in these contexts will determine whether an individual carries variants in both homologous copies of the gene, termed compound heterozygosity, or carries multiple variants in a single copy. The biological consequences of compound heterozygosity is exemplified by cancer predisposition syndromes involving deleterious germline alteration of the mismatch repair (MMR) genes [13]. Germline compound heterozygosity of MMR genes is associated with bi-allelic mismatch repair deficiency (bMMRD) and childhood onset cancer [14, 15], whereas mono-allelic germline altertion is associated with Lynch syndrome and adult onset cancer [16]. If a gene harbors multiple missense variants in a single copy, it is possible the combined effect of these variants on protein structure and function is different than the predicted independant effect of each variant. Further, non-coding eQTL variants can alter the expression of genes in an allele-specific fashion, modulating expression of the gene copy that lies on the same homologous chromosome [17, 18]. Determining which gene copy is under eQTL regulation can provide important information, particularly if one gene carryies inactivaing or dominant negative alleles [1]. Therefore, resolving the phase of both coding and non-coding variants can provide important insight into the biological consequences of germline variation.

We apply VAF phasing to 6180 whole exome sequencing (WXS) samples from the Cancer Genome Atlas (TCGA), and benchmark VAF phasing against two read-backed methods: HapCUT2 and phASER, one population-based method: SHAPEIT, and one laboratory-based method: 10X Genomics sequencing [19–22]. VAF phasing is highly concordant with all phasing methods assessed up to at distances of 10 Mb. We demonstrate the value of phase information by testing for association between germline variation in cancer predisposition genes and age of cancer diagnosis. We find suggestive evidence that carrying sets of non-compensatory missense variants in the same gene copy is associated with an earlier age of cancer diagnosis.

## Results

### Phasing with variant allele frequency

**Fig. 1:**
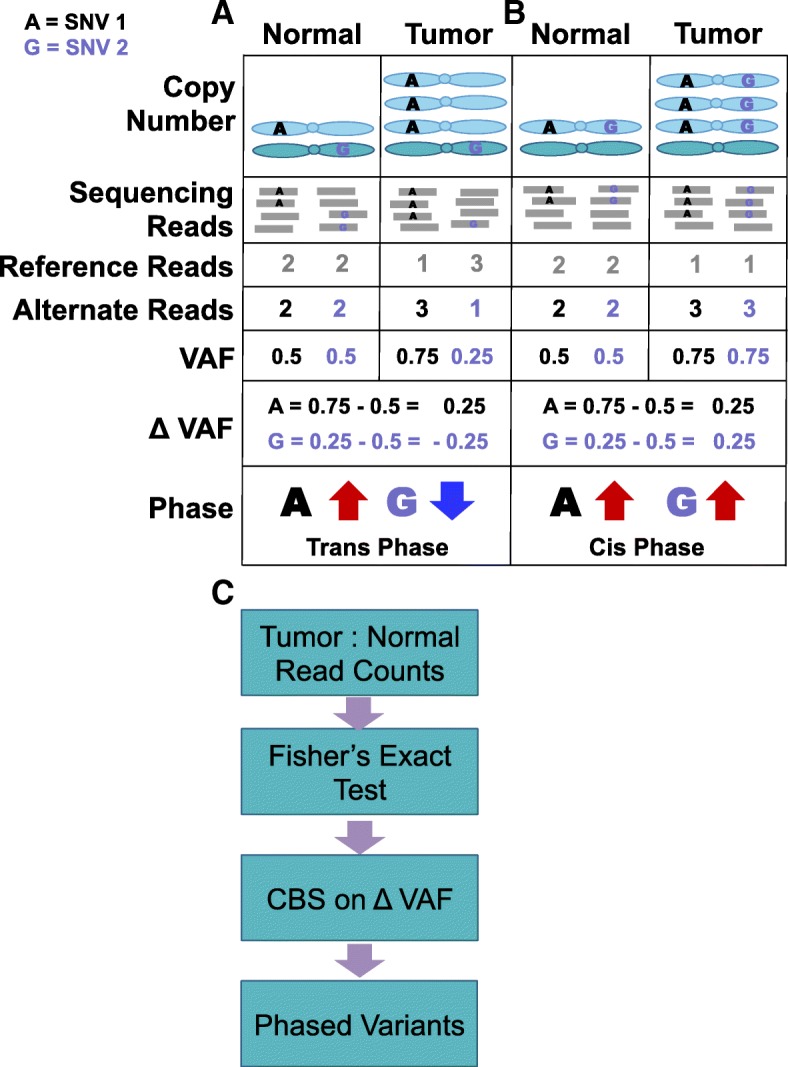
Overview of VAF phasing method. **a** The left panel illustrates two heterozygous germline SNVs in trans phase with the chromosome carrying SNV1 somatically amplified. In the normal sample, both SNVs have a VAF of 0.5. In the tumor sample, SNV1 is overrepresented in the sequence data (VAF = 0.75) and SNV2 is underrepresented (VAF = 0.25). The difference in VAF between the tumor and normal sample, which we refer to as *Δ* VAF, indicates that the VAF of SNV1 is increased (*Δ* VAF = 0.25) and that the VAF of SNV2 is decreased (*Δ* VAF = -0.25) in the somatic sample. For a pair of variants, somatic changes VAF in opposite directions suggest that the variants lie on different homologous chromosomes. **b** The right panel illustrates two heterozygous germline SNVs in cis phase with the chromosome carrying both SNV1 and SNV2 somatically amplified. In this case, both variants have an increased VAF (*Δ* VAF = 0.25). For a pair of variants, somatic changes VAF in the same direction suggest that the variants lie on the same homologous chromosome. **c** The VAF phasing pipeline has two steps: a Fisher’s exact test to identify sites with significant *Δ* VAF, and circular binary segmentation (CBS) on *Δ* VAF values to identify SCNA regions

It has been shown that somatic amplifications predominantly originate from a single germline homologous chromosome [6, 23]. Therefore, in SCNA regions of a tumor, one homologous chromosome is physically more abdundant than its partner. Due to this imbalance, sequencing reads will be skewed toward the more abundant homologous chromosome (Additional file 1: Figure S1). It follows that the somatic VAF of a heterozygous germline variant will deviate from the expected value of 0.5 in a SCNA region dependent which chromosome the variant lies on. Germline variants on the chromosome that is more abundant will appear to increase in VAF in the tumor, whereas variants on less abundant chromosomes will appear to decrease in VAF. Therefore, changes in VAF between tumor and normal samples, which we refer to as *Δ* VAF, can be used to infer chromosome of origin, or phase, of germline variants (Fig. 1a). Germline variants that lie on the same homologous chromosome (*cis* phase) will have *Δ* VAF values of similar magnitude and direction, whereas variants that lie on opposite homologous chromosomes (*trans* phase) will have *Δ* VAF values of similar magnitude but opposite direction. The VAF phasing method uses this simple intuition to phase variants and consists of two steps: 1) identify the coordinates of SCNAs 2) determine which heterozygous germline variants have significantly deviant *Δ* VAF to confidently phase and (Fig. 1b).

A number of methods exist to detect SCNAs from SNP array or NGS data, many of which use differences in signal intensity or read depth between normal and tumor samples to identify SCNA segments [5, 24]. Similarly, we reasoned that absolute *Δ* VAF could be used to identify SCNAs, as within a single SCNA the absolute *Δ* VAF of heterozygous germline variants should contiguous and of a similar magnitude (Additional file 1: Figure S2). While this approach does not distinguish amplifications from deletions, for the purposes of phasing germline variants only the coordinates of SCNAs are of interest. We applied circular binary segmentation (CBS), a method to partition the genome into segments with similar values, using absolute *Δ* VAF as input, a method we refer to as VAF-CBS [25]. While any SCNA calling method could be used to identify SCNAs coordinates for VAF phasing, we sought to provide a method that could be run entirely on paired tumor:normal reference and alternate read count data.

Identifying SCNA breakpoints using WXS data is difficult due to the sparse coverage of the genome, and this problem is exacerbated when only using heterozygous variants as informative data points [5]. In an effort to account for this known difficulty, we tested multiple values of a smoothing parameter that allows the CBS algorithm to join distant data points with similar *Δ* VAF values (see methods, Additional file 1: Figure S3). Increasing the smoothing distance resulted in longer predicted SCNA segments and more variants able to be phased overall (Additional file 1: Figure S3 and S4). However, msmoothing also carries the risk of missing SCNA breakpoints in regions not covered by exome capture. To balance the assumptions made by smoothing with the increased phasing capacity, we used a value of 1 Mb for future analyses.

**Fig. 2:**
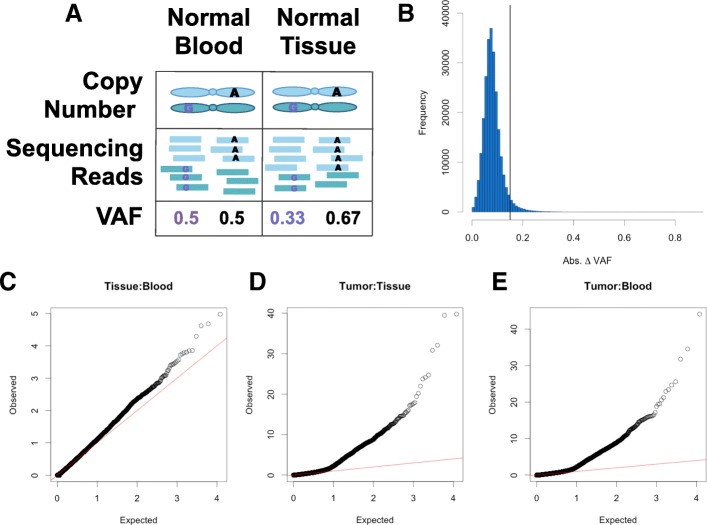
Using duplicated normal samples to identify SCNAs. **a** The expectation in diploid regions is that that the VAF of heterozygous SNVs will be 0.5; however, due to read sampling error, VAF greater or less than 0.5 is frequently observed. Duplicated normal samples in TCGA can be used as a null model to estimate how often read sampling error resembles an SCNA event by chance. **b** Distribution of mean segment absolute *Δ* VAF for 249,471 segments identified from *n*= 416 duplicated normal samples. Segments were identified using VAF-CBS with a smoothing parameter of 1 Mb. The solid line represents the 95% percentile (absolute VAF = 0.14). QQ plots showing p-values obtained from a Fisher’s exact test on tumor and normal read counts for an example sample **c** paired tumor:normal tissue, **d** tumor:normal blood, **e** normal tissue:normal blood

Changes in VAF between normal and tumor samples may be due to a biased read sampling, not a physical change in chromosomal copy number in the tumor. To determine a threshold to identify true SCNA segments above background noise, we utilized duplicated normal WXS samples. A subset of individuals in TCGA have multiple normal WXS samples, typically a blood and normal tissue sample. As there should be no CNAs in duplicated normal samples from the same individual, we used these samples to derive a null distribution for VCF-CBS (Fig. 2a). Interestingly, we identified seven duplicated normal samples with strong evidence of CNAs (Additional file 1: Figure S5). Given that the CNA regions observed in paired normal:normal samples were also observed in paired tumor:normal samples, we suspect this observation is due to tumor contamination of normal tissue and excluded these samples from further analysis (Additional file 1: Table S1). We ran VAF-CBS on duplicated normal samples from 416 individuals and observed 95% of segments identified have a mean absolute *Δ* VAF value < 0.14 (Fig. 2b). Therefore, we expect using a hard cutoff mean absolute *Δ* VAF of 0.14 to call SCNA segments would result in a 5% error rate. In an alternate approach, the mean absolute *Δ* VAF of each segment identified by VAF-CBS was compared to the mean absolute *Δ* VAF of the same genomic region in the duplicated normal samples, generating a null distribution for that specific genomic region. While this method has the advantage of accounting for region-specific read sampling noise, it is likely only applicable for samples within the TCGA cohort. We refer to these methods as “hard cutoff” and “region-specific” and use the region-specific null model for future analyses.

To identify variants with deviant *Δ* VAF, a Fisher’s exact test was performed for each germline heterozygous variant comparing reference and alternate read counts between tumor and normal samples. Only variants with a nominally significant *p*-value were considered for phasing. We used the duplicated normal samples mentioned above to confirm that the assumptions of the Fisher exact test were not violated. Indeed, the Fisher *p*-values for all heterozygous germline variants in duplicated normal samples followed the expected distribution, with a median 6% of heterozygous loci significant at a *p*< 0.05 cutoff (Fig. 2c). In contrast, a median 17% of heterozygous loci were significant in paired tumor:normal samples (Fig. 2d, e). By requiring that a variant both have a nominally significant *p*-value and be in a VAF-CBS SCNA region to be considered for phasing, we further reduce false positives due to read sampling noise. Applying VAF phasing with the hard cutoff null model to the duplicated normal samples, we observe only 0.3% of variants erroneously meet criteria for phasing.

### VAF phasing is concordant with other methods

We ran VAF phasing on 6180 TCGA samples using a range of smoothing parameters and both region-specific and hard cutoff null model approaches for SCNA identification. As there is no gold standard phasing dataset with paired tumor:normal sequence data, we assessed accuracy of our phase calls by comparing to TCGA germline phase calls generated by HapCUT2, phASER, and SHAPEIT (see methods) [19–21]. We observed a median 99% concordance between VAF phasing and both HapCUT2 and phASER (Additional file 1: Table S2). We observe the VAF method can phase on average 9% of variants phased by either HapCUT2 or phASER. Samples with poor concordance were largely those with few variants phased in common between methods (Additional file 1: Figure S6)). Choice of the smoothing parameter did not have a large effect on concordance; however concordance was lower when using the hard cutoff null model (Additional file 1: Table S2).To assess how robust our hard cutoff model concordance results were to changes in the mean absolute *Δ* VAF value parameter, we determined concordance between VAF phasing, HapCUT2, and phASER for mean absolute *Δ* VAF cutoff values from 0 - 0.6 (Additional file 1: Figure S7). We observe that higher cutoff values of mean absolute *Δ* VAF result in higher concordance, but fewer variants phased. Our chosen value of 0.14 aims to balance concordance with number variants phased.

**Fig. 3:**
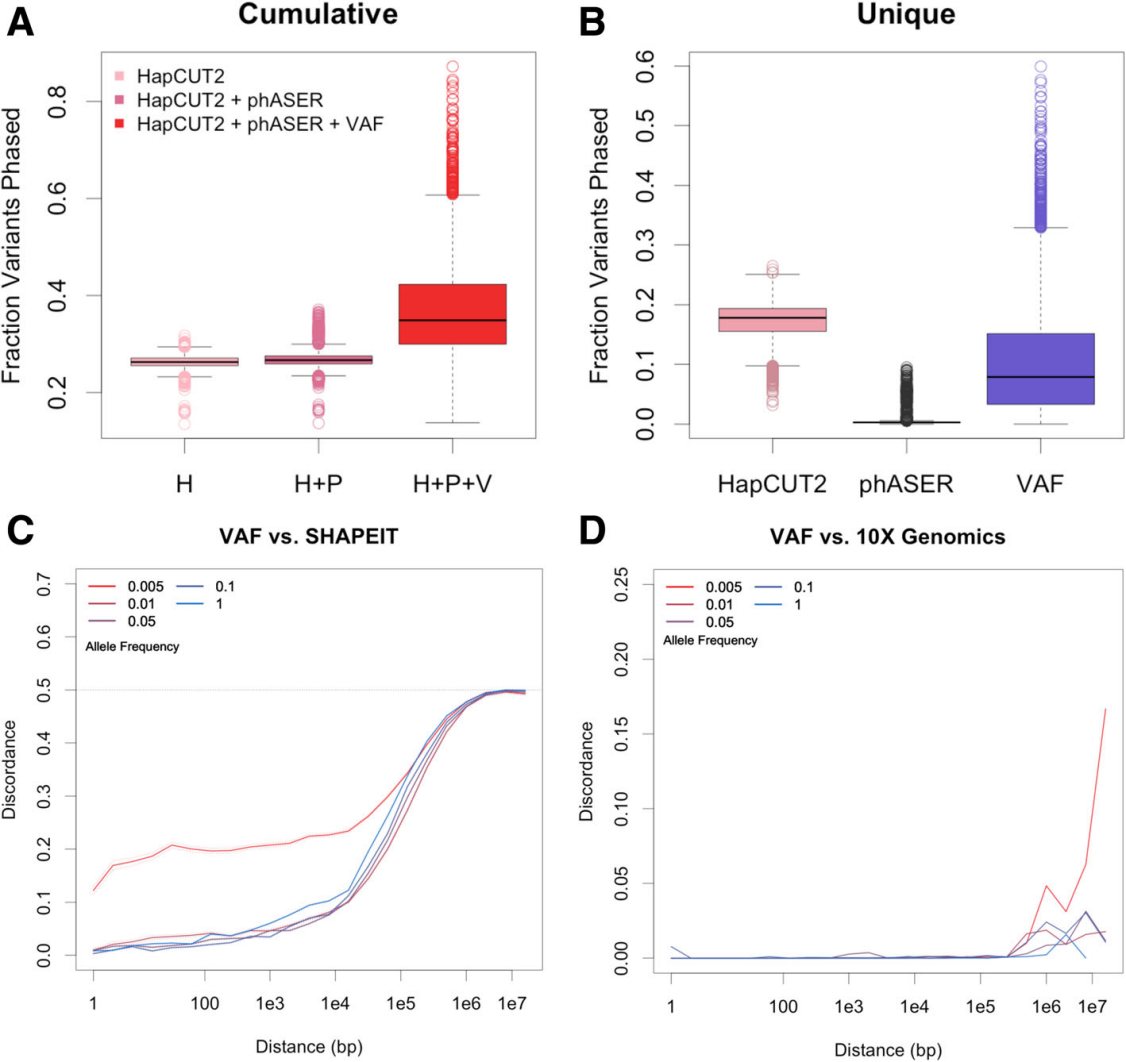
Comparison of phasing methods. Comparison of VAF phasing to read backed, population based, and laboratory phasing methods. **a** The fraction of germline heterozygous variants phased by HapCUT2 alone, HapCUT2 and phASER, and by HapCUT2, phASER, and VAF in *n*= 6180 samples. **b** The fraction of germline variants phased that are unique to each method. **c** Pairwise discordance between VAF phasing and SHAPEIT for *n* = 6263 samples as a function of distance and allele frequency. Pairs of variants were binned according to distance between the variants in base pairs and binned according to minimum allele frequency of the variant pair. Colors represent allele frequency bins. Solid lines represent the mean discordance, dotted lines are mean + = 2 s.e.m. **d** Pairwise discordance between VAF phasing and 10X Genomics phasing for the COLO829 cell line as a function of distance and allele frequency

There is considerable SCNA burden in TCGA samples, allowing VAF phasing to phase a median 1276 variants per sample (Additional file 1: Figure S8). The addition of VAF phasing to HapCUT2 and phASER increased the cumulative number of variants phased by 33% on average, and VAF phasing phased a median 942 variants not accessible to other methods (Fig. 3a,b). We observed similar results when restricting to rare variants (Additional file 1: Figure S9). The number of variants phased by VAF phasing is variable between samples and across genomic regions, consistant with the SCNA-dependant nature of the method (Additional file 1: Figure S10). We performed linear regression to identify factors underlying the performance of VAF phasing and found that the number of variants phased is largely determined by CNV burden and estimated tumor sample purity (Additional file 1: Table S3). We compared the performance of VAF phasing using SCNA calls derived from VAF-CBS vs. SCNA calls from TCGA SNP6 array data. A median 63% of variants were phased using both methods of SCNA identification (Additional file 1: Figure S11). However, the variants uniquely phased using VAF-CBS had higher concordance with HapCUT2 and phASER, suggesting that VAF-CBS identifies SCNA segements that produce more accurate phase calls (Additional file 1: Table S4).

We measured long range phasing performance using SHAPEIT phase calls and a pairwise approach to measuring phase accuracy (see methods, Additional file 1: Figure S12). VAF phasing and SHAPEIT are highly concordant up to approximately 10 kb (Fig. 3c). At distances larger than 10kb discordance between VAF phasing and SHAPEIT sharply increases, likely due to the fact that median haplotype block size in humans is 45 kb [26]. As expected, discordance was also higher for very rare and singleton variants, which are not amenable to phasing using population-based methods. To validate VAF phasing in a separate dataset, we compared VAF phasing to 10X Genomics phasing in COLO829, a tumor:normal pair of cell lines generated from a melanoma patient [22, 27]. Overall concordance between VAF phasing and 10X Genomics phasing was 99.23% (Additional file 1: Table S5). Pairwise discordance was largely unaffected by allele frequency and distance up to approximately 10 Mb (Fig. 3d). Supporting our previous finding that choice of VAF-CBS smoothing parameter doesn’t significantly impact phasing accuracy, we observe that discordance between VAF phasing and 10X Genomics phasing is similar for smoothing values between 0.5-2 Mb (Additional file 1: Figure S13, Table S5). Finally, to assess what genomic features of variants are associated with VAF phasing errors, we examined all phased variant pairs in the COLO829 sample. We observe that distance between variants is the feature most strongly associated with phase errors (Additional file 1: Table S6). This suggests that VAF phasing is less reliable at longer distances, which can potentially be ameliorated using WGS data to determine SCNA breakpoints.

### Application of VAF phasing to cancer predisposition

**Fig. 4:**
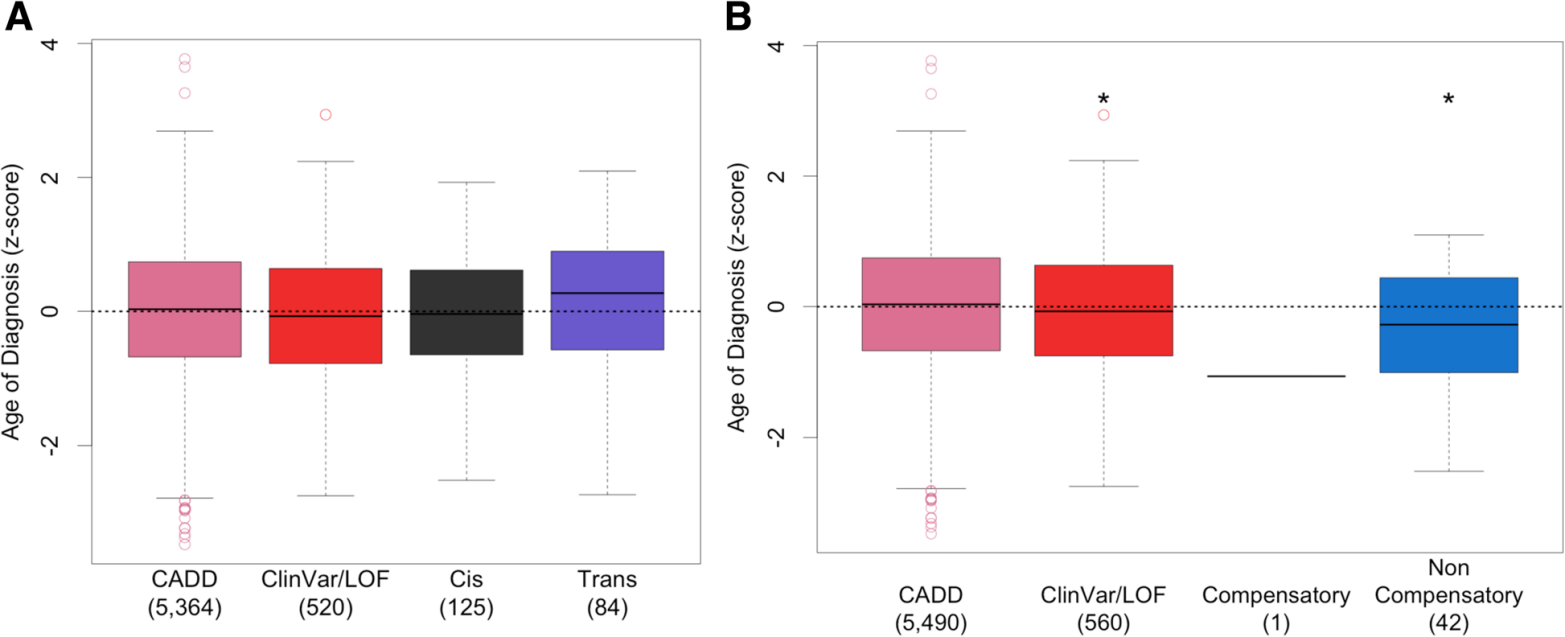
Leveraging Phase to Identify Cancer Predisposing Germline Variation. Association between germline compound heterozygosity events and non-compensatory cis variants and age of diagnosis. **a**-**b** Age of cancer diagnosis Z-score in *n*= 6093 TCGA individuals grouped by type of germline alteration in a set of 144 cancer predisposition genes. **a** Individuals were grouped into four hierarchical mutually exclusive groups based on compound heterozygosity status: those carrying a compound heterozygosity event (Trans), those with two or more phased CADD damaging variants in the same gene copy (Cis), those carrying mono-allelic ClinVar pathogenic or loss-of-function variants (ClinVar/LOF), and those carrying mono-allelic CADD damaging variants (CADD). **b** Individuals were grouped using HMMvar cis variant scores: those carrying a non-compensatory variant set (Non-Compensatory), those carrying a compensatory variant set (Compensatory), those carrying mono-allelic ClinVar pathogenic or loss-of-function variants (ClinVar/LOF), and those carrying mono-allelic CADD damaging variants (CADD). The number of samples is shown in parentheses. * = *p*< 0.05; *p*-values were determined using a linear model to predict age of diagnosis while accounting for cancer type

In genes that carry multiple variants, phase information disambiguates whether a single copy or both copies of the gene are altered. To demonstrate the value of phasing for biological analysis, we performed a phase-informed analysis relating germline variants in a set of 114 cancer predisposition genes to age of cancer diagnosis [12]. We first identified compound heterozygosity events, which we defined as carrying a variant with a CADD score ≥ 15 in both copies of a gene [28]. Using all read backed phasing methods combined, we were able to phase resolve 50% of all possible compound heterozygosity events exome-wide, and identified a total of 54,284 compound heterozygosity events in 4873 genes (Additional file 1: Figure S14). As we found few compound heterozygosity events for any single gene, we categorized individuals into four hierarchical mutually exclusive groups based on type of alteration in the predisposition gene set: those carrying a compound heterozygosity event (Trans), those with two or more phased CADD damaging variants in the same gene copy (Cis), those carrying mono-allelic ClinVar pathogenic or loss-of-function variants (ClinVar/LOF), and those carrying mono-allelic CADD damaging variants (CADD). We found no association between carrying a compound heterozygosity event in a cancer predisposition gene and age of cancer diagnosis (Fig. 4a, Additional file 1: Table S7).

We next asked whether carrying multiple missense variants in a single gene copy may be more deleterious than carrying a single variant. Variant scoring tools such as CADD scores are not designed to address this question, as they score variants independently [28]. Instead we used HMMvar, a variant scoring tool that assesses the collective effect of multiple missense variants and identifies sets of variants that collectively have a different score than expected based on single variant scores [29]. HMMvar identifies both compensatory variant sets, which collectively are less damaging than independently, and non-compensatory variant sets, which collectively are more damaging than independently. Similar to the previous analysis, we categorized individuals into four hierarchical mutually exclusive groups based on type of alteration in the predisposition gene set: those carrying a non-compensatory variant set (Non-Compensatory), those carrying a compensatory variant set (Compensatory), those carrying mono-allelic ClinVar pathogenic or loss-of-function variants (ClinVar/LOF), and those carrying mono-allelic CADD damaging variants (CADD). We found a significant association between carrying a non-compensatory variant set in a predisposition gene and an earlier age of cancer diagnosis (Fig. 4b, Additional file 1: Table S8). However, this may be in part due to six individuals who carry both a non-compensatory variant set and a ClinVar/LOF germline variant in different predisposition genes. Removing these samples reduces this association below nominal significance (Additional file 1: Figure S15, Table S9). *BRCA1/2* is one of the most frequently studied cancer predisposition genes [13, 30, 31]. Limiting analysis to *BRCA1/2* identified three non-compensatory variant sets and a suggestive, but not significant, association between carrying a *BRCA1/2* non-compensatory variant set and earlier age of diagnosis (Additional file 1: Figure S16, Table S10). Interestingly, all variants in the predicted *BRCA1/2* non-compensatory variant sets are individually predicted to be benign in ClinVar (Additional file 1: Table S11). Our results are suggestive that multiple missense variants that appear benign based on individual variant scores may collectively contribute to cancer predisposition.

## Discussion

A major assumption of VAF phasing is that SCNAs detected using VAF-CBS originate from a single germline homologous chromosome. This assumption is based off previous work showing somatic amplifications are predominantly mono-allelic [6, 23]. If VAF-CBS SCNA calls represent copy number alterations of similar magnitude from both homologs, phase switch errors will occur (Additional file 1: Figure S17). Given our high concordance with 10X Genomics long-range phasing, we believe our mono-allelic SCNA assumption is largely valid. Due to the sparse genomic coverage of the WXS data used in this study, we primarily apply VAF phasing to phase variants within a single gene. However, VAF phasing could be applied to paired tumor:normal whole genome sequencing (WGS) data, such as the 2800 WGS samples in PCAWG, to potentially phase up to entire chromosomes [32]. While the goal in developing VAF phasing was to create a straightforward and highly specific method, our model could be improved to incorporate uncertainty and increase sensitivity. Heterozygous variants in SCNA regions with a non-significant Fisher’s exact test could be given an estimated phase confidence score based on read count and population haplotype data. From a sample preparation perspective, VAF phasing could be improved by better isolation of tumor from normal tissue and with deeper sequencing depth.

VAF phasing can be used to extract more value from the numerous existing paired tumor:normal datasets. Phasing germline variants from individuals with cancer is not only of interest to understanding cancer predisposition, as we demonstrated, but also to population genetics as a whole. Phased germline variants obtained from cancer data can serve as a reference dataset of phased gene haplotypes. The human leukocyte antigen (HLA) locus is of great importance to many diseases, including autoimmune diseases, infection, and cancer [33]. The complex nature and high degree of polymorphism of this region makes phasing difficult [34]. In the TCGA samples we examined, 3651 individuals have SCNA of chromosome 6p spanning the major histocompatibility locus (MHC) region, including 602 ethnically-diverse samples. VAF phasing could potentially be incorporated into existing HLA phasing methods to facilitate phasing of this region and increase the knowledge base of known HLA haplotypes. HLA typing of cancer patients has become increasing important in personalized immunotherapy [35]. VAF phasing could potentially also be leveraged for patients with chromosome 6 SCNA to better estimate individual HLA haplotypes.

Using phase information we identified compound heterozygosity events and sets of missense variants in the same homologous gene copy predicted to negatively interact. We found no significant association between carrying a compound heterozygosity event in a cancer predisposition gene and age of cancer diagnosis; however, we found that individuals carrying non-compensatory missense variant sets had a significantly earlier age of diagnosis. While it seems counterintuitive that alteration of both gene copies is less deleterious than alteration of a single copy, it’s likely that our definition of compound heterozyosity included missense variants that don’t fully disrupt gene function. Using a more strict threshold to identify damaging variants, we observe few individuals carrying compound heterozygosity events, presumably because dual inactivation of cancer predisposing genes would result in childhood onset cancer [14] (Additional file 1: Figure S18). We noted that different predisposition genes were preferentially affected by compound heterozygosity vs. those affected by non- compensatory missense events, which may be in part due to selection against dual alteration of specific genes key for survival. Further, using VAF phasing we are unable to resolve all possible compound heterozygosity events, thus we likely underestimated of the effect of compound heterozygosity.

We identified 42 missense variant sets in 20 predisposition genes predicted to have a non-compensatory effect on protein function. We investigated non-compensatory *BRCA1/2* missense variants in detail and noted that all were independently annotated as benign in ClinVar. This could indicate that there is a negative interaction between variants or that some of the variants are miss-annotated as benign in ClinVar. While there are a tremendous number of tools aimed at predicting the functional effect of individual missense variants, few methods exist that predict the effect of multiple missense variants simultaneously [28, 29, 36, 37]. There is some evidence in cardiovascular disorders that multiple missense variants in a gene are more deleterious than single variants [38]. However, as it is not routine to assess the potential for negative interactions between multiple missense variants in a gene, the importance is likely underestimated. High throughput in vitro assays have been used to predict the effect of 2000 amino acid substitutions on *BRCA1* E3 ubiquitin ligase activity [39]. Similar approaches could be used to assess the joint effect of multiple missense variants in a protein.

## Conclusions

In this study we present a simple method to phase germline variants in preexisting tumor:normal sequencing datasets. We demonstrate VAF phasing is highly concordant with two read-backed and one laboratory-based phasing method, and that the addition of VAF phasing to existing read-backed methods increased the number of variants phased by an average of 33%. VAF phasing performs well on common and rare variants and at long distances, with the potential to phase up to entire chromosome lengths with WGS data. We identified individuals from TCGA that carry multiple missense variants in a single gene copy predicted to collectively be more deleterious than independently, and show that carrying one of these non-compensatory variant sets in a cancer predisposition gene is associated with an earlier age of cancer diagnosis. Our work demonstrates the biological relevance of phasing germline variants in cancer and highlights the need for better scoring tools to account for multiple variants in a single

## Methods

### Data acquisition

Approval for access to TCGA case sequence and clinical data were obtained from the database of Genotypes and Phenotypes (project #8072: Integrated analysis of germline and somatic perturbation as it relates to tumor phenotypes). WXS germline variant calls from 8542 individuals were obtained using GATK v3.5 as described previously [40]. Samples prepared using whole genome amplification (WGA) were excluded from analysis due to previous identification of technical artifacts in both somatic and germline variant calls in WGA samples [40]. Raw somatic WXS sequence data and somatic RNA-seq data was downloaded from the legacy archive of the genomic data commons (GDC) in BAM file format aligned to the hg19 reference genome [41]. Segmented SNP6 array data were downloaded from Broad Firehose (release stddata__2016_01_28, file extension: segmented_scna_hg19). Aggregate allele frequencies and allele frequencies in 7 ancestry groups (African, Admixed American, East Asian, Finnish, non-Finnish European, South Asian, and other) were obtained from ExAC v3.01 [42]. Clinical biospecimen histology slide data for tumor purity measurements was downloaded from GDC.

### Variant annotation and filtering

Raw variant calls were filtered using GATK VQSR TS 99.5 for SNVs and TS 95.0 for indels. Putative germline loss-of-function (LOF) variants were identified using the LOFTEE plugin for VEP and Ensembl release 85 [43]. Only germline LOF variants with an AF < 0.05 in all ancestry groups represented in ExAC were used in the age of diagnosis association analyses. Gene, CADD score, and ClinVar annotations were obtained using ANNOVAR and ClinVar database v.20170905 [44]. A germline variant was determined to be pathogenic using ClinVar annotations if at least half of the contributing sources listed the variant “Pathogenic” or “Likely Pathogenic”.

### Implementation of VAF phasing

Somatic reference and alternate read counts for germline variants were obtained from the germline VCF and somatic BAM files using samtools mpileup v1.3.1 (SNPs) or varscan v2.3.9 (indels) [45, 46]. Germline variants not present in the somatic sequence data were excluded from further analysis. A two-way Fisher’s exact test comparing reference and alternate read counts was performed on all germline variants to test for deviation in VAF between the normal and tumor sample. Only sites with a nominally significant (*p*< 0.05) change in VAF between tumor and normal sample were considered for phasing. Circular binary segmentation (CBS) was performed on absolute *Δ* VAF values, calculated as abs(somatic VAF- germline VAF), of all heterozygous germline variants using the R package ’PSCBS’, a process we refer to as VAF-CBS [25]. Smoothing of gaps between heterozygous sites was implemented using the function ‘findLargeGaps’ and setting ‘minLength’ to the values of 0.5, 1, 2, or 3 Mb. For all segments containing > 1 variants, the mean absolute *Δ* VAF of all variants was calculated. Significant segments were determined either directly using a region-specific or a hard cutoff null model. In the region-specific model, the absolute *Δ* VAF of each segment identified by VAF-CBS is compared to the absolute *Δ* VAF of the same genomic region in *n*=416 paired normal replicate samples. Segments with an absolute *Δ* VAF in the 90th percentile were considered significant and to represent true SCNA. In the cutoff model, a hard cutoff of absolute *Δ* VAF ≥ 0.14 was used to identify significant segments. Within each SCNA segment, variants with a nominally significant Fisher’s exact test were assigned to a chromosome of origin using the sign of *Δ* VAF, such that all variants with an increasing VAF are assigned to one chromosome and all variants with a decreasing VAF are assigned to the other. For analyses using TCGA SCNA calls, segments with an estimated fold change value < 0.9 or > 1.1, which corresponds to a single chromosome loss or gain in 20% of tumor cells, were considered significant. VAF phasing was applied to a total of 6180 TCGA samples with tumor WXS, normal WXS, somatic RNA-seq, and evidence of SCNA burden > 0.

### 10X genomics phasing

Germline WGS data phased using 10X Genomics technology and paired tumor:normal WXS data from COLO829 was obtained from Jonathan Keats and his lab at TGEN in the form of VCFs [22, 27]. VAF phasing was performed as previously described on the paired tumor:normal WXS data. To create a set of variants to use to compare VAF phasing with 10X Genomics phasing, the WGS data was limited to coding regions +/- 100 bases at each exon boundary. The WGS data was further filtered to only include variants with a minimum 20X read depth in the germline WXS sample and at least 2X read depth in the somatic WXS sample.

### Comparison to other phasing methods

HapCUT2 was run with default parameters using germline WXS BAM files from GDC and single sample VCFs of germline variant calls generated as described previously [21]. PhASER was run with the parameters –mapq 255, –baseq 10, and –paired_end 1 [19]. The HLA region was blacklisted with the –blacklist option and indels were excluded from analysis. Phaser was run on somatic RNA-seq BAM files and single sample germline VCFs. For SHAPEIT phasing, the germline VCF from the full cohort of 8542 individuals from TCGA was converted to PLINK bed format, excluding multiallelic sites [20]. SHAPEIT was run with default parameters on the full cohort with the genetic HapMap phase II recombination map provided by SHAPEIT specified with the -M parameter.

To determine overall discordance between two methods, phase blocks in common between both methods were found. Within a common block, the number of variants with disagreeing phase orientation by the two methods as well as the total number of variants phased in common were counted. Discordance was calculated as: 1 - (the number of concordant phased variants/number of phased variants in common) (Additional file 1: Figure S12). To obtain pairwise discordance and features of individual phase pairs, all unique pairwise combinations of variants were identified within each common phase block. Pairs with disagreeing phase orientation were considered discordant (Additional file 1: Figure S12). Additional features calculated for each phase pairs were: Minimum Read Depth = lowest read depth of phase pair, Segment Size = size of VAF-CBS segment in base pairs, Segment Abs. *Δ* VAF = absolute *Δ* VAF of the VAF-CBS segment, *Δ* *Δ* VAF = difference in *Δ* VAF between the phase pair variants, Pair Distance = distance between phase pair variants in base pairs, *Δ* Allele Frequency = difference in allele frequency between phase pair variants, Minimum Allele Frequency = lowest allele frequency of phase pair variants.

### HMMvar annotation and compound heterozygosity analysis

HMMvar v.1.1.0 was used to jointly assess the functional effect of multiple cis-phased nonsynonymous variants [29]. HMMvar is a method that uses a hidden markov model computed from multiple sequence alignment of homologous proteins to predict the effect of multiple nonsynonmyous coding variants in a gene based on amino acid conservation of the variant set. For each gene and each individual, a gene variant set was constructed using phased heterozygous and homozygous nonsynonymous variants. For each gene, the RefSeq standard transcript or the longest coding transcript was used to calculate HMMvar scores. HMMvar scores were calculated for individual variants and for variant sets. Compensatory variant sets were defined as those with a set score ≤ min (individual variant scores) - 1.5 * (max (individual variant scores) - min (individual scores)), non-compensatory variant sets were defined as those with a set score ≥ max (individual variant scores) + 1.5 * (max (individual variant scores) - min (individual scores)).

For identifying compound heterozygosity events, variants with a CADD score ≥ 15 were considered damaging. Compound heterozygosity events were defined at the gene level as possessing two damaging variants in trans configuration (one variant in each copy). Cis damaging events were defined as possessing two damaging variants in cis configuration (two variants in one copy). For compound heterozygosity analyses that used phase calls from all read backed phasing methods, phased variants were combined as follows. For variants with VAF phasing, the VAF phasing calls were used. For variants without VAF phasing, HapCUT2 or phASER phase calls were used. For variants with both HapCUT2 and phASER phase calls, phase calls were only included if HapCUT2 and phASER agreed.

### Statistical analyses

Principal Component Analysis (PCA) was performed on common (AF > 0.01) germline variants using PLINK v1.90b3.29 and the first two principal components obtained from this analysis were used to control for ancestry in all of the regression models we fit to the data [47]. To test for association between germline alteration and age of diagnosis a linear model of the form A ~G _ij_ + X _i_ was used where A denotes age of diagnosis, G _ij_, is a binary indicator for germline alteration status of gene j in sample i, and X _i_ represents a vector of covariates for sample i (cancer type, PC1, PC2).

## Additional file

Additional file 1 This file contains supplemental Tables S1-S11, and supplemental Figures S1-S16. (PDF 3482 kb)

## Abbreviations

bMMRD: Bi-allelic mismatch repair deficiency
CBS: Circular binary segmentation
GDC: Genomic data commons
HLA: Human leukocyte antigen
LOF: Loss-Of-function
MHC: Major histocompatibility locus
NGS: Next generation sequencing
PCA: Principal component analysis
SCNA: Somatic copy number alteration
TCGA: The cancer genome atlas
VAF: Variant allele frequency
WGA: Whole genome amplification
WGS: Whole genome sequencing
WXS: Whole exome sequencing
