# Supplementary material for: Rare variant phasing using paired tumor:normal sequence data
Source: BMC Bioinformatics. 2019 May 27;20:265. doi: 10.1186/s12859-019-2753-1 (PMC6537421; doi:10.1186/s12859-019-2753-1)
Supplement: Supplementary file 1 — This file contains supplemental Tables S1-S11, and supplemental Figures S1-S16. (PDF 3482 kb) [file 12859_2019_2753_MOESM1_ESM.pdf]

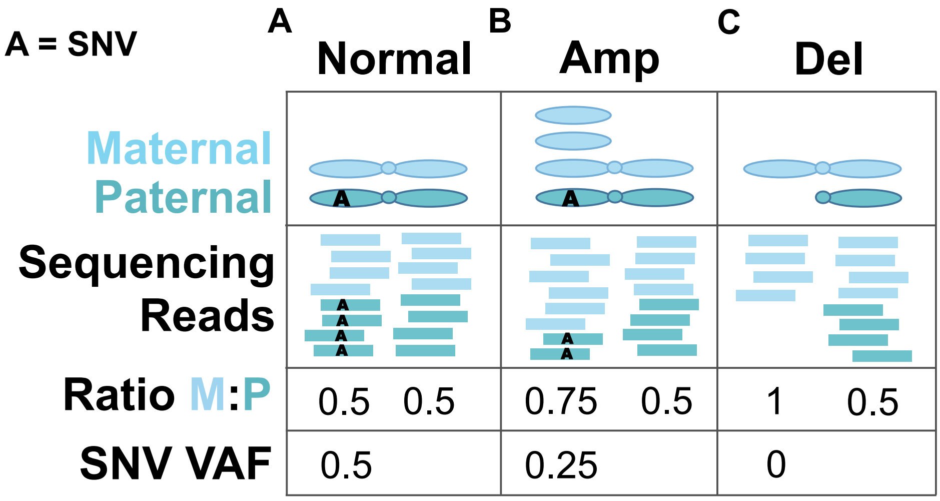

Figure 1: In regions of SCNA, somatic sequencing reads are skewed toward the chromosomes that are physically more abundant in the sample. Here we illustrate the maternal chromosome in blue and the paternal in green. (A) The expectation in diploid regions is that the sequencing reads will originate from the maternal and paternal chromosome in a 0.5 ratio and that the VAF of heterozygous SNVs will be 0.5. (B) The expectation in regions of amplification is that sequencing reads will be skewed toward the amplified chromosome and that the VAF of heterozygous germline SNVs will change from 0.5. In this illustration the SNV lies on the non-amplified chromosome and therefore the VAF decreases to 0.25. (C) The expectation in regions of deletion is that sequencing reads will be skewed toward the non-deleted chromosome. In this illustration the SNV lies on the deleted chromosome and therefore the VAF decreases to 0.

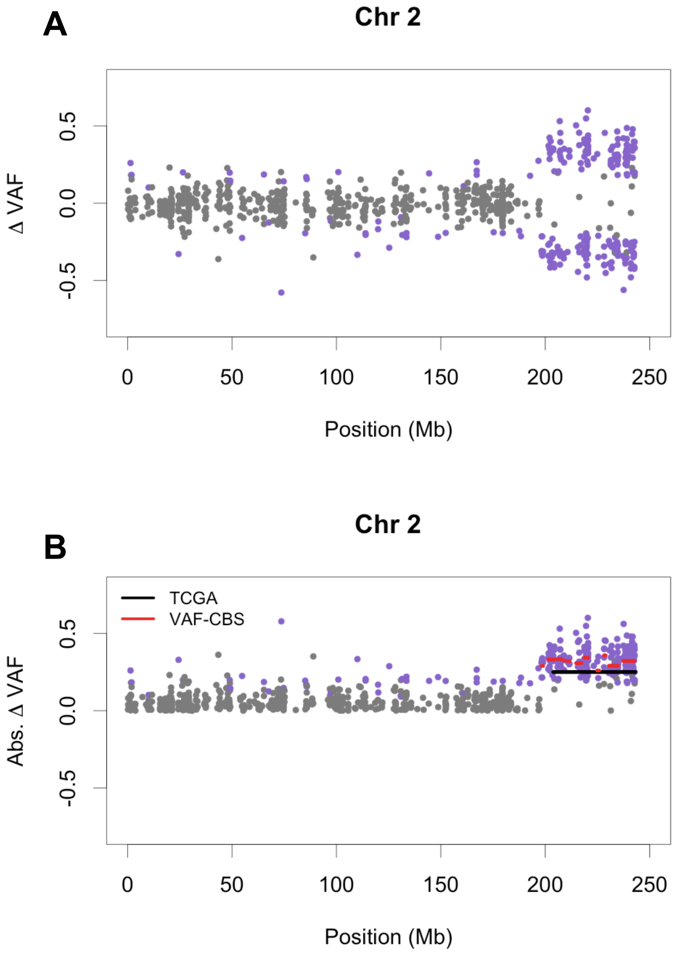

Figure 2: Example data illustrating Δ VAF changes in SCNA regions for chromosome 2 of sample TCGA-Y8-A8RZ. (A) Δ VAF for 812 germline heterozygous variants. (B) Absolute Δ VAF for the same variants as (A). SCNA segments identified using TCGA data are shown as black lines, SCNA segments identified using VAF-CBS are shown in red. Color indicates p-value obtained from a Fisher's exact test on tumor and normal read counts: gray: $p \geq 0.05$, purple: $p < 0.05$.

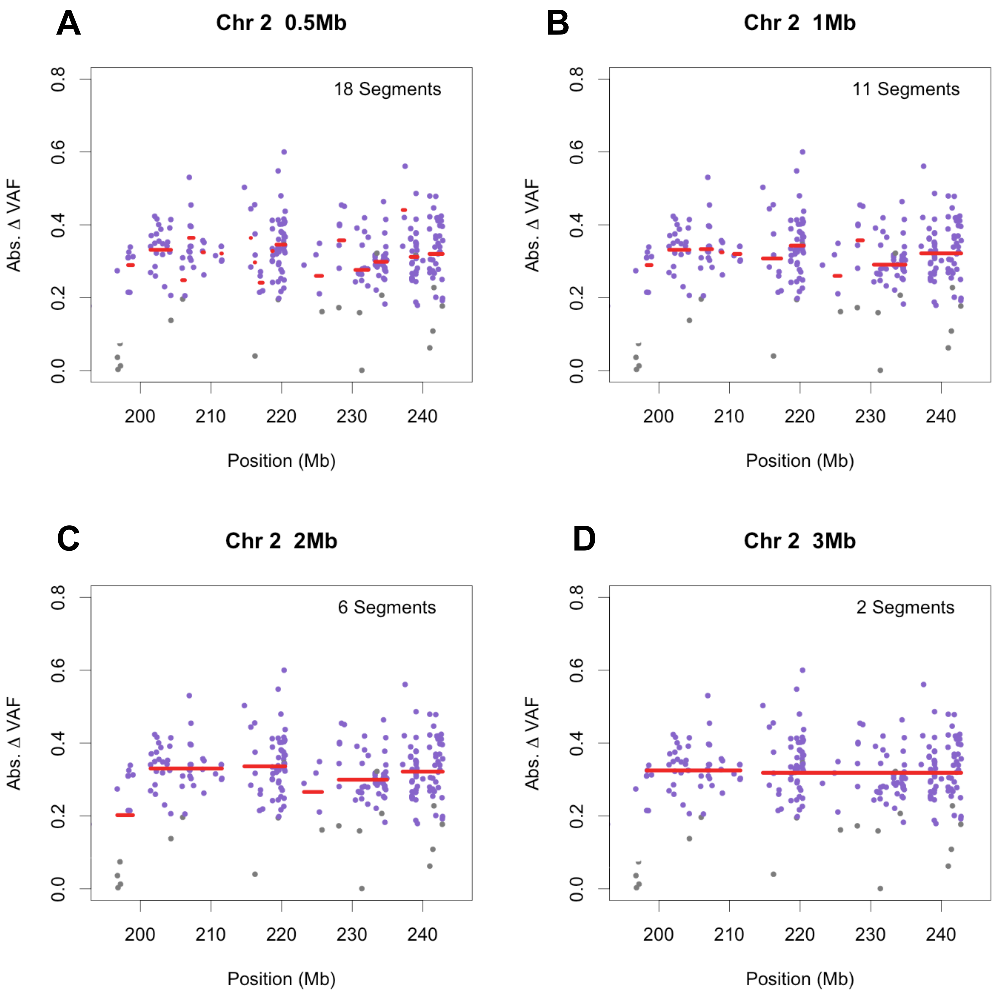

Figure 3: Example data illustrating VAF-CBS segments identified using different smoothing parameters for a SCNA region in chromosome 2 of sample TCGA-Y8-A8RZ. VAF-CBS segments are shown as red lines and the total number of segments in the region is shown in the upper right corner. Color indicates Fisher exact test p-value using tumor and normal read counts: gray: $p \geq 0.05$, purple: $p < 0.05$. Four different smoothing parameters were tested: (A) 0.5 megabases (Mb), (B) 1 Mb, (C) 2 Mb, (D) 3 Mb.

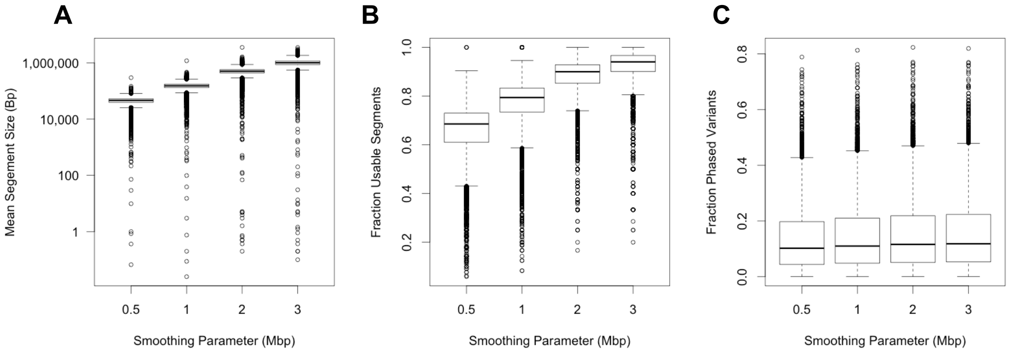

Figure 4: SCNA segment metrics from $n = 6{,}158$ samples using four different VAF-CBS smoothing parameters: 0.5 Mb, 1 Mb, 2 Mb, and 3 Mb. (A) Mean size in base pairs of segments identified. (B) Fraction of segments identified that contain more than one heterozygous variant. (C) Fraction of all germline heterozygous variants that can be phased.

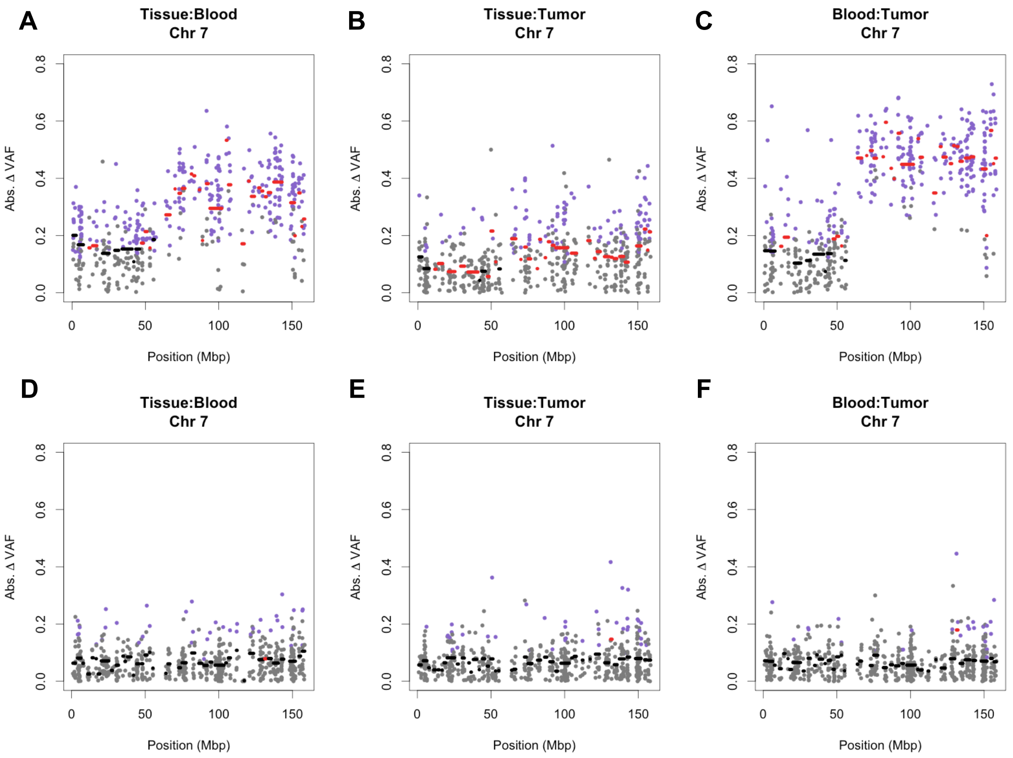

Figure 5: Example data illustrating absolute Δ VAF changes in (A) paired normal tissue:normal blood, (B) normal tissue:tumor, and (C) normal blood:tumor for chromosome 7 of sample TCGA-V5-AASX. Example data illustrating absolute Δ VAF changes in (D) paired normal tissue:normal blood, (E) normal tissue:tumor, and (F) normal blood:tumor for chromosome 7 of sample TCGA-Y8-A8RZ. Color indicates p-value obtained from a Fisher's exact test on tumor and normal read counts: gray: $p \geq 0.05$, purple: $p < 0.05$. Segments with an absolute Δ VAF ≥ 0.14 are red.

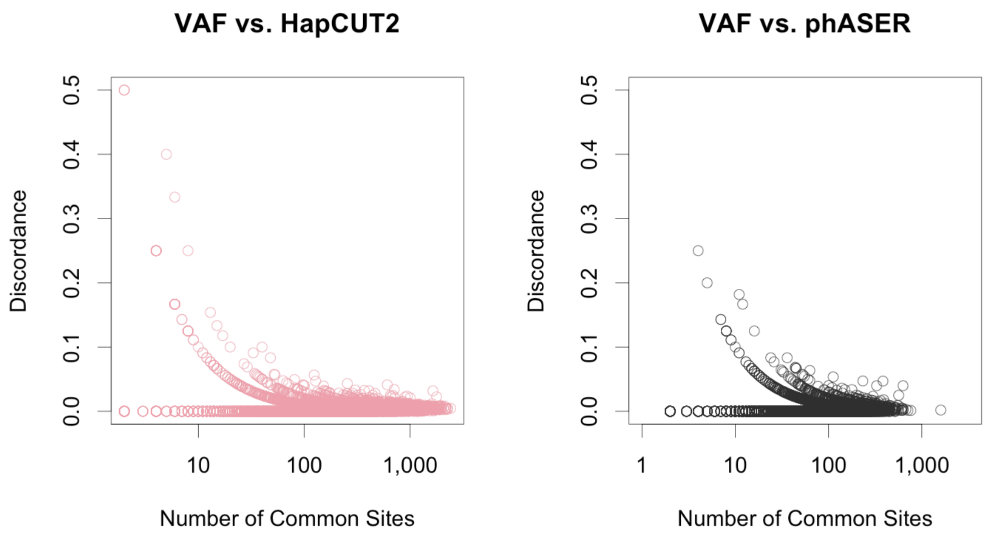

Figure 6: Discordance between phasing methods. (A) Discordance between VAF phasing and HapCUT2 for n = 6,180 samples. Number of common sites are number of sites phased by both methods. (B) Discordance between VAF phasing and phASER for n = 6,180 samples.

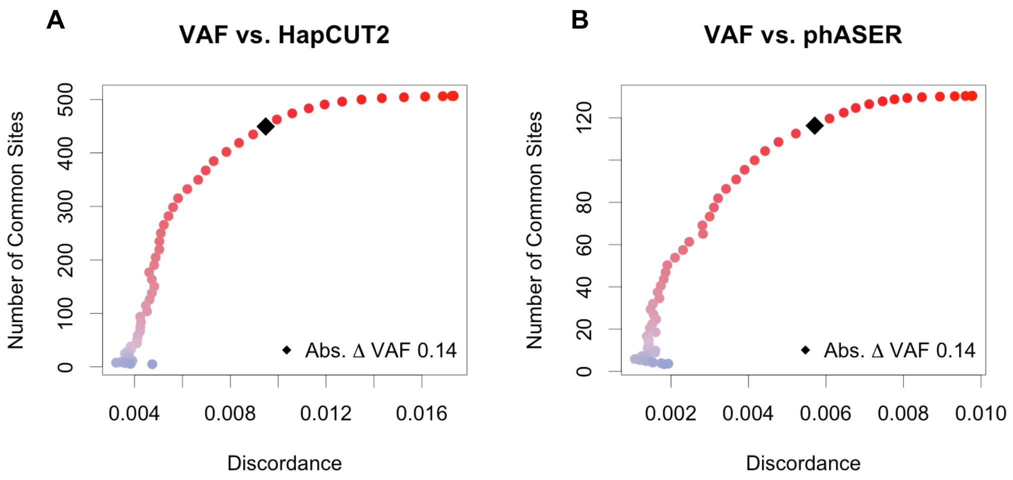

Figure 7: Tuning the Mean Absolute Δ VAF Cutoff Value for the Hard Cutoff Δ VAF Model. Mean discordance vs. mean number of variants phased in common between VAF phasing and (A) HapCUT2 or (B) phASER for values of mean absolute Δ VAF ranging from 0 - 0.6 in n = 6,000 samples.

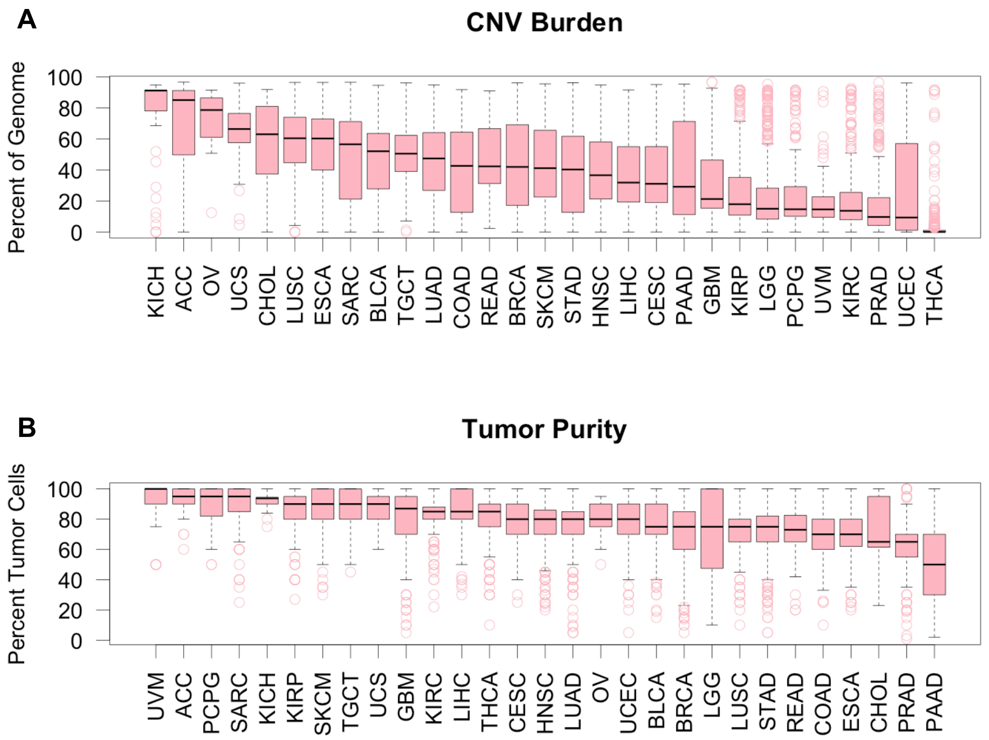

Figure 8: Sample metrics that affect ability to call phase using Δ VAF. (A) Percent of the genome involved in a SCNA event for n = 8,542 TCGA samples across 29 cancer types. Fraction of the genome calculated as: total length in base pairs of TCGA SCNA regions (predicted FC > 1.1 or < 0.9) / $3e^{09}$ (B) Percent tumor nuclei for the same samples as (A). Percent tumor nuclei values were obtained from TCGA biospecimen histology slide data. Samples with missing values were imputed to cancer type median.

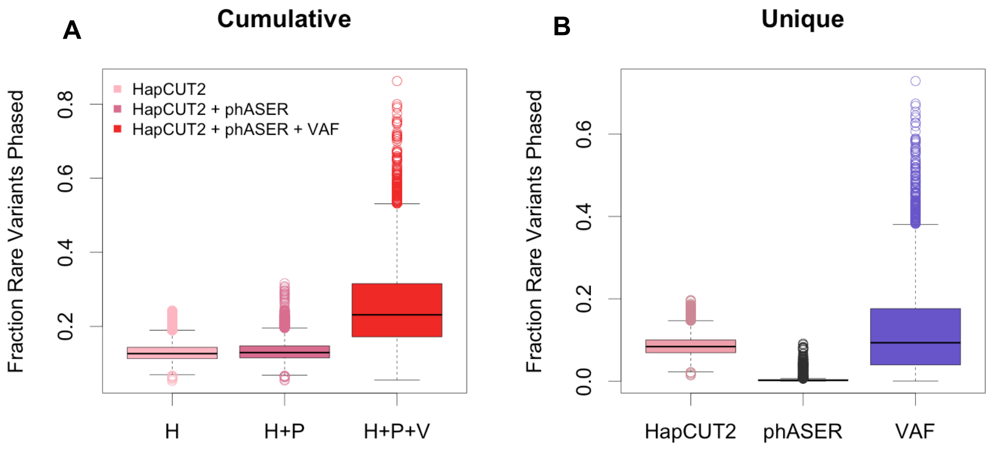

Figure 9: (A) The fraction of rare (allele frequency ≤ 0.01) germline heterozygous variants phased by HapCUT2 alone, HapCUT2 and phASER, and by HapCUT2, phASER, and VAF in $n = 6{,}180$ samples. The addition of VAF phasing increased the number of phased variants by 98%. (B) The fraction of rare germline variants phased that are unique to each method.

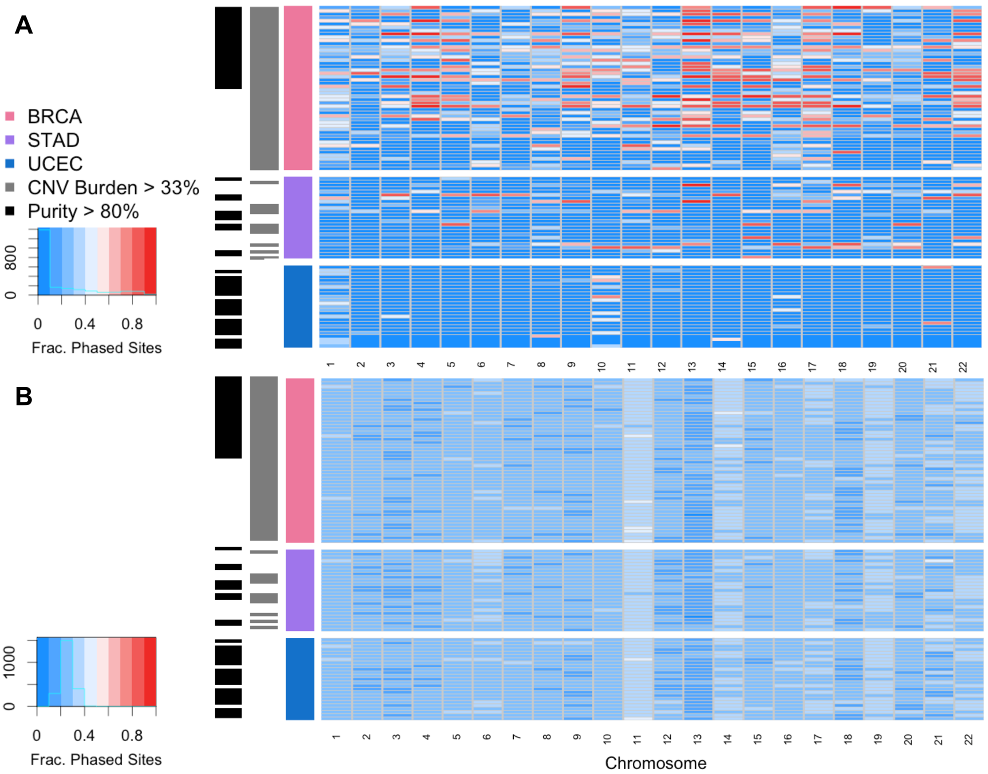

Figure 10: The fraction of phased variants visualized by chromosome. (A) Fraction of heterozygous germline variants phased using VAF phasing for n = 100 samples. Cancer type is indicated by color: BRCA = Breast Invasive Carcinoma, STAD = Stomach Adenocarcinoma, UCEC = Uterine Corpus Endometrial Carcinoma. Samples with more than 33% of the genome involved in an SCNA are indicated by gray bars. Samples with a histology slide based purity > 80% are indicated by black bars. (B) Fraction of heterozygous germline variants phased using HapCUT2 for the same samples as (A).

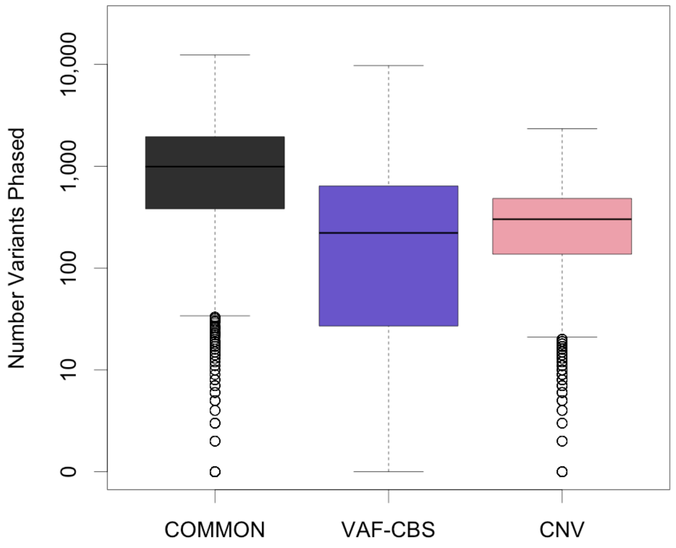

Figure 11: Comparing number of variants phased using TCGA SCNA segments vs. VAF-CBS segmentation. COMMON = variants phased by both methods, VAF-CBS = variants phased only using VAF-CBS segmentation, CNV = variants phased only using TCGA CNV calls. A median 63% of variants were phased by both methods in n = 6,180 samples.

A

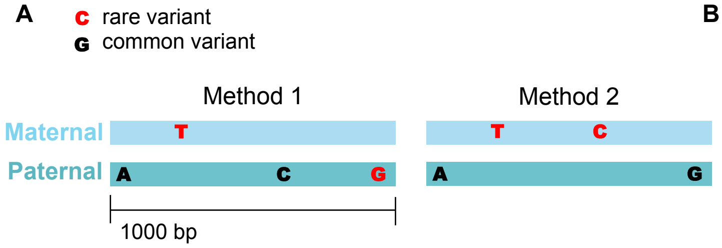

B

Phase 1	Phase 2
1|0	1|0
0|1	0|1
**1|0**	**0|1**
1|0	1|0
**Overall Discordance: 0.25**	

C

Pair	Distance	Minimum Allele Frequency	Mismatch
A : T	100	0.01	0
A : C	700	0.5	1
A : G	1000	0.01	0
T : C	600	0.01	1
T : G	900	0.01	0
C : G	300	0.01	1

D

Distance	AF	Pairwise Discordance
< 500	< 0.1	0.5
>= 500	< 0.1	0.33
< 500	> 0.1	NA
>= 500	> 0.1	1

Figure 12: Method used to calculate pairwise error. (A) Example of a shared phase segment showing phase calls from two methods. Rare variants (allele frequency ≤ 0.01) are shown in red. (B) The overall discordance was calculated as the fraction of discordant phase calls within a block. (C) Table showing all pairwise phase pairs from the segment in (A). Distance between pairs is calculated as distance in base pairs between the variants. Minimum allele frequency is the smaller allele frequency of the two variants. Error is a binary variable that indicated whether the two variants are in the same orientation. (D) Example showing how pairwise error was binned by distance and allele frequency as in Figure 3. For each category the mean error was calculated.

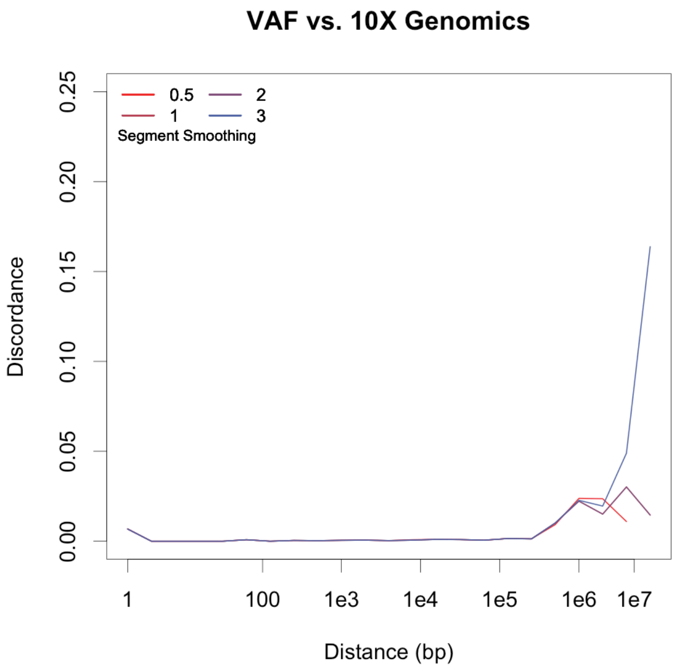

Figure 13: Pairwise discordance between VAF phasing and 10X Genomics phasing for the COLO829 cell line as a function of distance and allele frequency. Colors represent different values of the smoothing parameter.

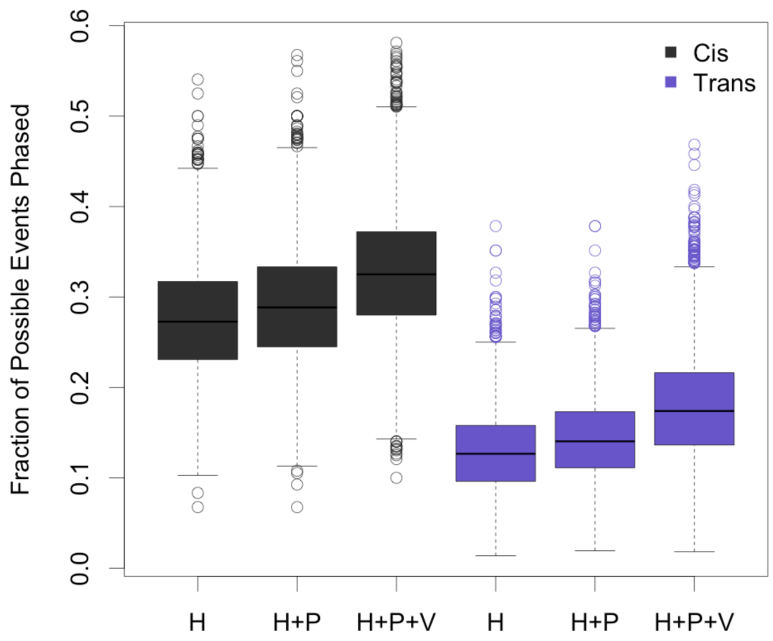

Figure 14: Fraction of genes with possible compound heterozygosity events phased. Fraction was calculated for each individual as: the number of genes with multiple CADD $\geq$ 15 variants phased / the number of genes with multiple CADD $\geq$ 15 germline variants. H = HapCUT2 only, H+P = HapCUT2 and phASER, H+P+V = HapCUT2, phASER, and VAF phasing.

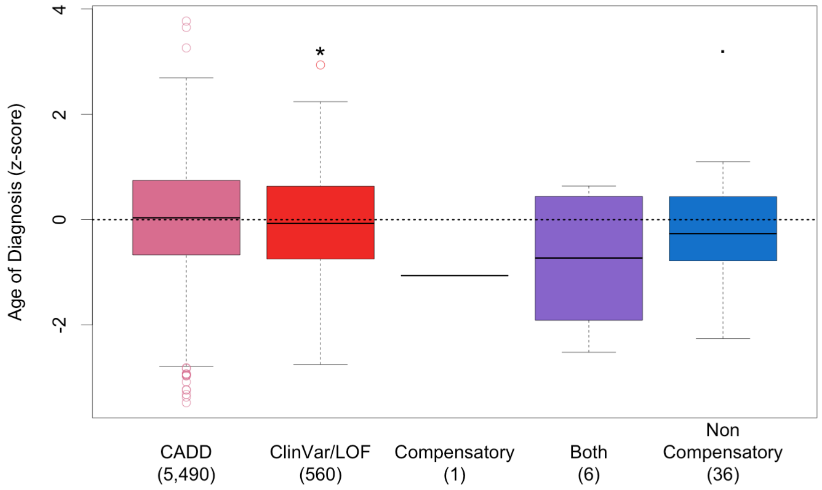

Figure 15: Age of cancer diagnosis Z-score in n = 6,093 TCGA individuals grouped by type of germline alteration in a set of 144 cancer predisposition genes. Groups are the same as Figure 4B with the exception of six individuals carrying both a non-compensatory variant set and a ClinVar/LOF variant that are grouped separately (Both). * = $p < 0.05$, . = $p < 0.1$; p-values were determined using a linear model to predict age of diagnosis while accounting for cancer type.

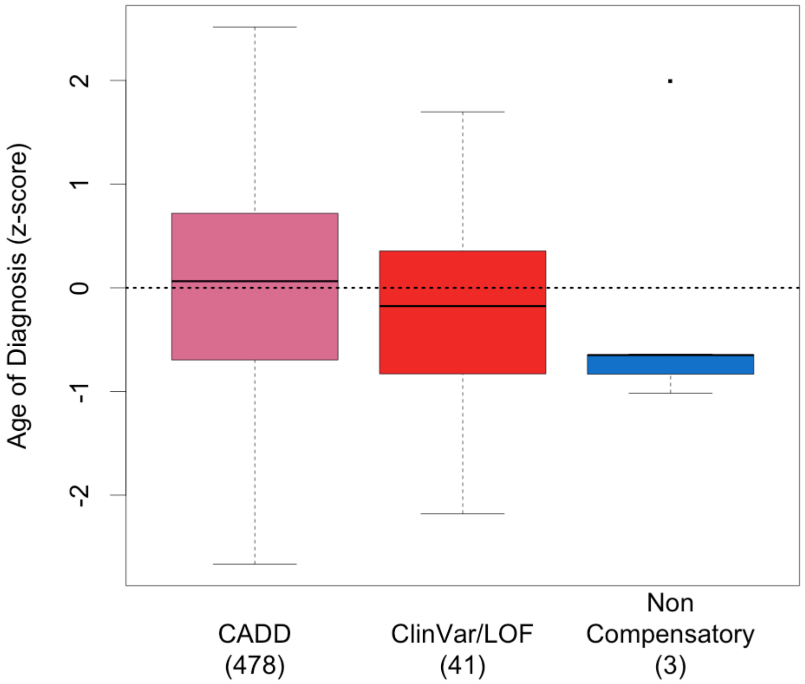

Figure 16: Age of cancer diagnosis Z-score in 6,093 TCGA individuals grouped by type of germline alteration in *BRCA1* or *BRCA2*. (B) Individuals were grouped using HMMvar cis variant scores: CADD = individuals carrying a germline variant with a CADD score ≥ 15, ClinVar/LOF = individuals carrying a ClinVar pathogenic or LOF germline variant, Non-Compensatory = individuals carrying multiple nonsynonymous variants in a gene predicted to be more deleterious collectively than independently The number of samples is shown in parentheses. * = $p < 0.05$, . = $p < 0.1$; p-values were determined using a linear model to predict age of diagnosis while accounting for cancer type.

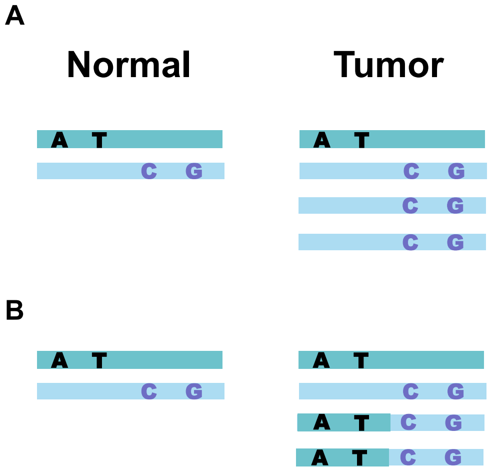

Variant	VAF	Δ VAF	Abs. Δ VAF	Phase
A	0.25	-0.25	0.25	1|0
T	0.25	-0.25	0.25	1|0
C	0.75	0.25	0.25	0|1
G	0.75	0.25	0.25	0|1

Variant	VAF	Δ VAF	Abs. Δ VAF	Phase
A	0.75	0.25	0.25	0|1
T	0.75	0.25	0.25	0|1
C	0.75	0.25	0.25	0|1
G	0.75	0.25	0.25	0|1

Figure 17: VAF phasing assumes that SCNA segments originate from a single homologous chromosome. (A) Under this assumption, Δ VAF can be used to correctly phase variants. (B) Should a SCNA segment result from equal amplification of both homologous chromosomes, using Δ VAF to phase will result in switch errors. In this example, amplification of both homologous chromosomes results in all variants being assigned to the same chromosome incorrectly.

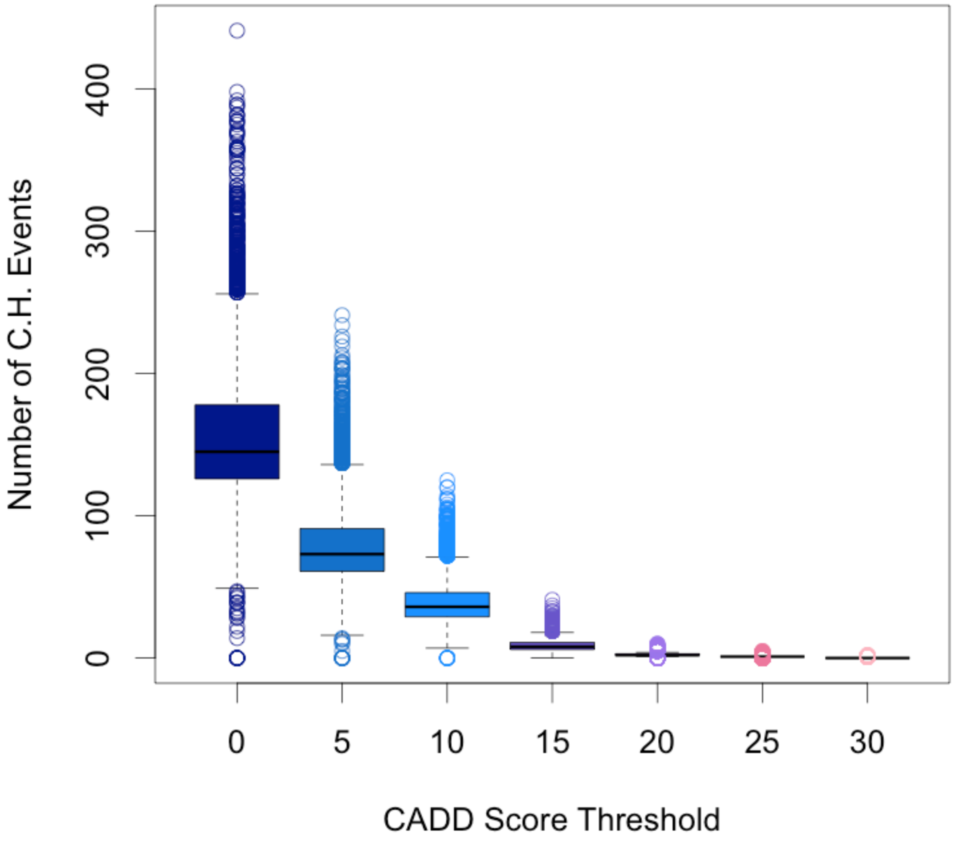

Figure 18: Using CADD Scores to Identify Compound Heterozygosity. The number of compound heterozygosity events identified in n = 6,093 TCGA individuals using a CADD score threshold ranging from 0 - 30.

Table 1: TCGA barcodes of suspected contaminated normal tissue samples.

Contaminated Normal Sample Barcodes
TCGA-4G-AAZT
TCGA-BH-A0BQ
TCGA-BH-AB28
TCGA-BR-6710
TCGA-G9-6362
TCGA-V5-A7RE
TCGA-V5-AASX

Table 2: Factors that influence number of variants phased per sample by VAF phasing.

		**Region Specific**				**Cutoff**			
**Phase Comparison**	**Smoothing Parameter (Mb)**	**Mean Disc.**	**Std. Error**	**Mean Num. Sites**	**Std. Error**	**Mean Disc.**	**Std. Error**	**Mean Num. Sites**	**Std. Error**
VAF : HapCUT2	$5.00e^{+05}$	0.00321	$1.04e^{-04}$	374.13	4.499	0.00740	$2.65e^{-04}$	388.25	4.638
VAF : HapCUT2	$1.00e^{+06}$	0.00358	$9.78e^{-05}$	386.04	4.596	0.00654	$2.64e^{-04}$	383.62	4.636
VAF : HapCUT2	$2.00e^{+06}$	0.00374	$9.76e^{-05}$	395.21	4.655	0.00517	$1.85e^{-04}$	381.066	4.632
VAF : HapCUT2	$3.00e^{+06}$	0.00400	$1.32e^{-04}$	400.03	4.679	0.00513	$2.23e^{-04}$	379.66	4.629
VAF : phASER	$5.00e^{+05}$	0.00237	$1.38e^{-04}$	91.63	1.201	0.00406	$1.53e^{-04}$	97.367	1.249
VAF : phASER	$1.00e^{+06}$	0.00269	$1.55e^{-04}$	95.38	1.233	0.00395	$1.50e^{-04}$	96.302	1.251
VAF : phASER	$2.00e^{+06}$	0.00289	$1.04e^{-04}$	97.84	1.250	0.00371	$1.42e^{-04}$	95.527	1.250
VAF : phASER	$3.00e^{+06}$	0.00302	$1.10e^{-04}$	98.81	1.257	0.00388	$1.62e^{-04}$	95.223	1.251

Table 3: Discordance between VAF phasing and other phasing methods. Null model refers to VAF phasing using duplicated samples directly to determine significant VAF-CBS segments, cutoff refers to using a absolute Δ VAF cutoff of 0.14 to determine significant VAF-CBS segments.

	**Beta**	**Std. Error**	**t value**	**P**
**Purity**	0.00665	0.000297	22.339	$2.60e^{-106}$
**CNV Burden**	0.0122	0.000201	60.676	0.0
**Tumor Depth**	-0.000305	0.000712	-0.428	0.669
**Normal Depth**	0.000504	0.000620	0.813	0.416
**Depth Ratio**	0.000675	0.0504	0.0134	0.989

Table 4: Discordance between VAF phasing and other phasing methods using VAF-CBS vs. TCGA SCNA calls Mean discordance between VAF phasing, HapCUT2 and phASER in n = 6,180 samples either using VAF-CBS segments or TCGA SCNA calls.

**Segmentation**	**Phase Comparison**	**Mean Discordance**	**Std. Error**
VAF-CBS	VAF : HapCUT2	0.0062	$4.92e^{-04}$
VAF-CBS	VAF : phASER	0.0029	$2.37e^{-04}$
TCGA CNV	VAF : HapCUT2	0.0321	$5.89e^{-04}$
TCGA CNV	VAF : phASER	0.0173	$6.10e^{-04}$

Table 5: Discordance between VAF phasing and 10X Genomics phasing in COLO829.

Smoothing Parameter (Mb)	Fraction Variants Phased	Number Variants Phased	Number of Errors	Discordance
5.00E+05	0.354	12103	79	0.00652
1.00E+06	0.363	12435	95	0.00763
2.00E+06	0.363	12428	95	0.00764
3.00E+06	0.367	12568	168	0.0133

Table 6: Features of incorrect VAF phasing pairs from COLO829. Features were calculated for all possible pairs of phased heterozygous germline variants from COLO829 (see Methods).

Feature	Correct Pairs Mean	Incorrect Pairs Mean	% Difference (correct - incorrect)	Wilcox p-value
Minimum Read Depth	314.5993	284.0293	-10.7629	$< 2.2e^{-16}$
Segment Size (bp)	15,317,251	11,160,745	-37.2442	$< 2.2e^{-16}$
Segment Abs. Δ VAF	0.1637	0.1687	2.9418	$< 2.2e^{-16}$
Δ Δ VAF	0.1560	0.1591	1.8939	$< 8.093e^{-10}$
Pair Distance (bp)	1,209,932	2,021,657	40.1514	$< 2.2e^{-16}$
Δ Allele Frequency	0.2180	0.2582	15.5696	$< 2.2e^{-16}$
Minimum Allele Frequency	0.2509	0.2968	15.4387	$< 2.2e^{-16}$

Table 7: Association between germline compound heterozygosity status and age of cancer diagnosis.

	Beta	Std. Error	t value	p
ClinVar/LOF	-1.33	0.565	-2.352	0.0187
Cis	-0.812	1.11	-0.731	0.464
Trans	1.64	1.35	1.212	0.225

Table 8: Association between germline non-compensatory cis variants and age of cancer diagnosis.

	Beta	Std. Error	t value	p
ClinVar/LOF	-1.25	0.5.46	-2.288	0.0221
compensatory	-12.0	12.3	-0.978	0.328
non compensatory	-4.34	1.90	-2.281	0.0225

Table 9: Association between germline non-compensatory cis variants and age of cancer diagnosis with confounding samples removed.

	Beta	Std. Error	t value	p
ClinVar/LOF	-1.250	0.545	-2.29	0.0220
Compensatory	-12.002	12.268	-0.978	0.327
Both	-8.128	5.013	-1.621	0.105
Non-Compensatory	-3.710	2.054	-1.806	0.071

Table 10: Association between germline non-compensatory cis variants in *BRCA1/2* and age of cancer diagnosis.

	Beta	Std. Error	t value	p
ClinVar/LOF	-1.199	1.975	-0.607	0.544
non compensatory	-12.88	6.99	-1.843	0.065

Table 11: Germline non-compensatory cis variants in *BRCA1/2*.

Indv	Cancer	Chr	Pos	Ref	Alt	AA	Variant	CADD	ExAC AF	ClinVar	Detail
A	KIRC	17	41246481	T	C	p.Q309R	Miss	9.735	0.04407	B	
A	KIRC	17	41246662	T	C	p.R249G	Miss	16.08	8.24E-06	conflicting	likely benign 1, VUS 2
B	UCS	17	41245071	G	T	p.T779K	Miss	11.61	0.0001813	B	
B	UCS	17	41246662	T	C	p.R249G	Miss	9.735	0.04407	B	
C	HNSC	13	32906729	A	C	p.N372H	Miss	6.228	0.2779	B	
C	HNSC	13	32911278	T	C	p.L929S	Miss	8.566	0.0008861	B	
C	HNSC	13	32911452	A	T	p.N987I	Miss	9.104	0.0008728	B	
